# Copper Complexes: Emerging Micro- and Nanosystems for Dermatological Treatment

**DOI:** 10.3390/pharmaceutics18070784

**Published:** 2026-06-26

**Authors:** Ireri Hernández-Rojas, Javier Aguila-Rosas, Oswaldo Castañeda Hernández, Carlos Martínez-Armenta, Verónica Barón-Flores, Betzabeth A. García-Martínez, Camilo Rios

**Affiliations:** 1 Maestría en Ciencias Farmacéuticas, Universidad Autónoma Metropolitana, Calzada del Hueso 1100, Col. Villa Quietud, Mexico City 04960, Mexico; 2251802205@alumnos.xoc.uam.mx; 2 Laboratorio de Farmacia Molecular y Liberación Controlada, Departamento de Sistemas Biológicos, Universidad Autónoma Metropolitana, Calzada del Hueso 1100, Col. Villa Quietud, Mexico City 04960, Mexico; jaguila@correo.xoc.uam.mx; 3 Laboratorio de Fisicoquímica y Reactividad de Superficies (LaFReS), Instituto de Investigaciones en Materiales, Universidad Nacional Autónoma de México, Circuito Exterior s/n, CU, Del. Coyoacán, Mexico City 04510, Mexico; 4 Departamento de Sistemas Biológicos, Universidad Autónoma Metropolitana, Calzada del Hueso 1100, Col. Villa Quietud, Mexico City 04960, Mexico; ocastaneda@correo.xoc.uam.mx; 5 Laboratorio de Gerociencias, Instituto Nacional de Rehabilitación Luis Guillermo Ibarra Ibarra, Calz México-Xochimilco 289, Mexico City 14389, Mexico; c.armenta1208@gmail.com; 6 Laboratorio de Neurofarmacología Molecular, Departamento de Sistemas Biológicos, Universidad Autónoma Metropolitana, Calzada del Hueso 1100, Col. Villa Quietud, Mexico City 04960, Mexico; vbaron@correo.xoc.uam.mx; 7 División de Neurociencias, Instituto Nacional de Rehabilitación Luis Guillermo Ibarra Ibarra, Calz México-Xochimilco 289, Mexico City 14389, Mexico

**Keywords:** copper complex, nanostructure, microstructure, dermatological

## Abstract

The use of copper complexes as pharmacotherapy represents an emerging strategy with multiple therapeutic advantages, including their enhanced bioavailability, antimicrobial activity, and ability to participate in diverse cellular processes. These molecules, combined with micro- and nanosystems, offer an advanced approach to the sustained delivery of copper, optimizing its absorption while potentially reducing adverse effects. In this review, we highlight the application of copper complexes reported in recent studies for dermatological diseases and infection management. Furthermore, evidence indicates that copper promotes cell regeneration in wounds and burns, accelerating wound healing. However, their clinical translation requires careful consideration of copper homeostasis, as dysregulation may lead to oxidative stress and toxicity. In perspective, the combination of micro- and nanoformulations with specific copper complexes offers new opportunities for tailored therapies, as well as for the optimization of pharmacokinetics, positioning copper as a multifunctional therapeutic agent in regenerative and supplementation medicine. However, further investigation is required to establish safety, optimal dosing, and long-term effects.

## 1. Introduction

Metal ions such as iron [1], zinc [2], manganese [3], cobalt [4], and copper are important in the body due to their role in various processes as enzymatic cofactors [5], for example, in DNA synthesis and repair [6].

Copper is a trace element present in all human tissues and is essential for vital biochemical processes, such as in signaling pathways for glucose, cholesterol, and iron metabolism [7,8]. It also maintains cardiovascular integrity, lung elasticity, bone formation, and erythrocyte production [9]. Its bioavailability is estimated to range between 30 and 40% depending on the salt used. Copper salts are more common due to their simple structure and synthesis, as well as their easy dissociation in solutions [10]. Copper ions are absorbed in the stomach and the distal small intestine, subsequently entering enterohepatic circulation. This systemic distribution is primarily mediated by ceruloplasmin and albumin [11].

Some authors suggest that bioavailability may be significantly lower (around 10%) [9]; therefore, the use of modified release systems is crucial to overcome current therapeutic challenges. In humans, the tolerable upper level for copper is 10 mg/day, although one study suggests a daily intake of closer to 3 mg/day may be more appropriate for adults [12]. A balanced diet ensures the optimal functioning of copper in the body’s biochemical processes. However, an excess of this metal can lead to its accumulation in several tissues, particularly the liver and sometimes in the brain [13].

On the other hand, copper forms complexes with a wide range of organic molecules, enabling versatile interactions with different chemical species. Its electronic configuration allows for multiple coordination geometries and bonding modes [14,15]. This opens the possibility of finding new therapies that use copper complexes and their ions as catalytic or therapeutic agents. Consequently, multiple lines of research have emerged, focusing on obtaining and characterizing these compounds due to their active role in redox and biological processes, which gives them clinical potential in various pathologies. Recent studies show that the deregulation of copper homeostasis is related to metabolic diseases such as anemia [16], neutropenia [17] and other disorders caused by copper deficiency, as well as serious neurological disorders, including models of Alzheimer’s disease [18,19], amyotrophic lateral sclerosis [20,21] and Menkes disease [22,23], due to its impact on essential enzymes and neuronal function.

In this context, the use of copper complexes has been explored as a therapeutic strategy. For example, copper histidinate, a Cu^2+^ complex with histidine, is administered parenterally to improve copper absorption in patients with Menkes disease and has shown significant benefits when administered early after diagnosis [22,23]. This reflects the interest in modulating the bioactivity of copper through complexes to correct imbalances of this metal and mitigate its pathological consequences, an approach supported by various reviews that highlight the role of copper complexes in medicine and their potential for future therapeutic applications [24,25,26]. In turn, the potential use of copper complexes in the treatment of dermatological conditions [25,27,28] and, more recently, burn injuries [29,30] has also been investigated, showing promising results in terms of accelerating healing and modulating the local inflammatory response due to their biological effects on angiogenesis, tissue regeneration, and anti-inflammation after injury [31]. In addition, the use of copper complexes as antimicrobial agents to prevent wound infection [32,33] has emerged as a potential solution to one of the major problems associated with bacteria: antibiotic resistance [34].

Regarding their administration, several studies have explored the use of micro- and nanostructured systems, which constitute a modified delivery-and-release strategy for therapeutic molecules [35], as well as metal ions [36]. Due to their size, these systems facilitate distribution to specific tissues [37,38], while the use of polymers in their formulation allows for prolonged release [37].

This review analyzes the use of various copper complexes and their formulations in micro- and nanostructured systems designed for modified delivery and targeting different therapeutic targets in dermatological conditions. The reviewed studies highlight that these structures aim to improve the stability, bioavailability, and therapeutic efficacy of copper, in addition to modulating its release and reducing its potential toxic effects.

## 2. Copper Homeostasis: Systemic Regulation and Response to Supplementation

In the human body, copper is a metallic ion capable of participating in a wide variety of biochemical processes due to its ability to be reduced (Cu^1+^) or oxidized (Cu^2+^) in biological environments. It is important to highlight that the oxidation state of the metal defines the affinity of the metal ion for its ligands; while Cu^1+^ preferentially binds to amino acids such as cysteine and methionine through thiol or sulfide groups, Cu^2+^ shows higher affinity for acidic amino acids and histidine, interacting through oxygen atoms and imidazole nitrogens [39].

**Figure 1:**
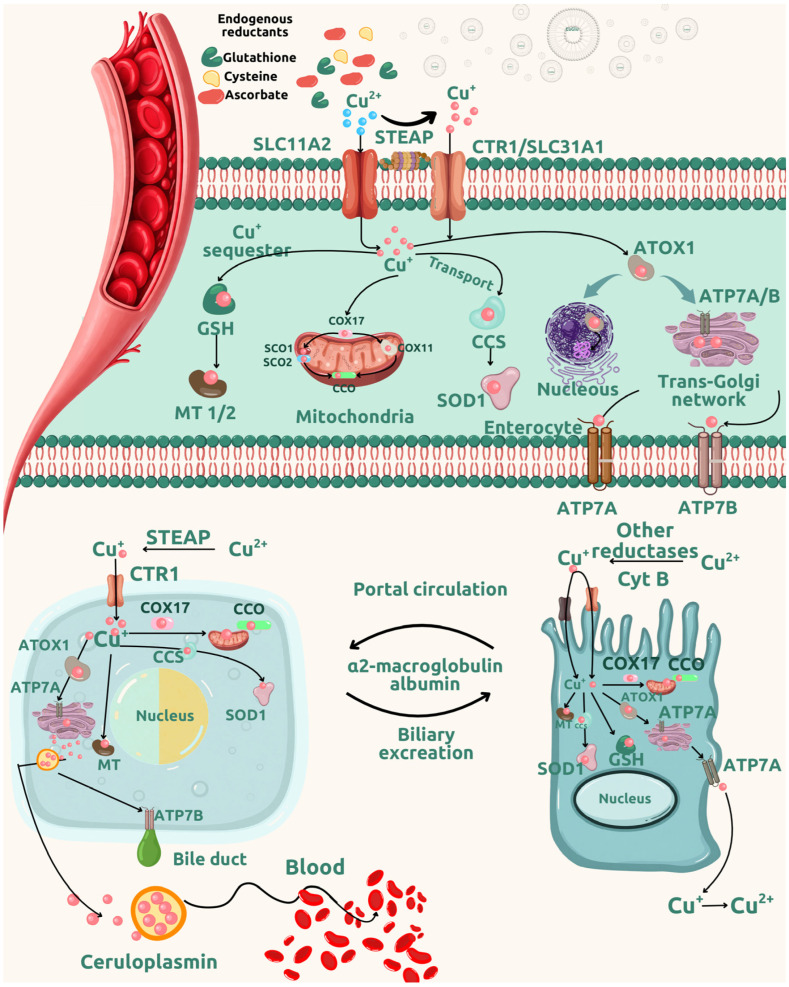
Schematic representation of copper metabolism and intracellular transport mechanisms in enterocytes and hepatocytes.

The main source of the metal is through diet, where it is found in its oxidized form (Figure 1). However, for the ion to be absorbed in the duodenum, it must be reduced to Cu^1+^. Copper transporter 1 (CTR1) is the main protein responsible for ion internalization, as it can bind Cu^2+^, which is subsequently reduced by weak biological reductants present in the environment, such as ascorbate [40], glutathione [41], and cysteine. Additionally, a family of proteins known as six-transmembrane epithelial antigen of the prostate (STEAP) [42] can reduce metal ions such as Fe^3+^ and Cu^2+^. Divalent metal transporter 1 (DMT1) is another protein capable of internalizing Cu^1+^ ions. In the cytosol, Cu^1+^ ions are sequestered by chaperone molecules such as cytochrome c oxidase copper chaperone (COX17), copper chaperone for superoxide dismutase (CCS), and antioxidant protein 1 (ATOX1), which donate the metal ion to cytochrome c oxidase, the Zn/Cu-dependent superoxide dismutase enzyme (Zn/Cu SOD), and the trans-Golgi network, respectively. The following metal efflux pumps are located in this organelle: ATPase 7A (ATP7A), which is responsible for redistributing the metal by delivering it to cuproproteins, and ATPase 7B (ATP7B), which mediates the elimination of excess metal via the biliary route [43].

Copper is distributed through plasma as exchangeable Cu^2+^ (an ion available for metabolic functions), mainly bound to two molecules: albumin and transcuprein [44]. These proteins allow for distribution to the rest of the body’s tissues, as they can directly interact with CTR1 and donate Cu^2+^ [40]. In contrast, the distribution of non-exchangeable copper (Cu^1+^) is regulated by ceruloplasmin (Cp), an enzyme that requires copper as a cofactor for its catalytic activity and is responsible for transporting approximately 75% of plasma copper. It is also associated with low-molecular-weight molecules and the Zn/Cu SOD enzyme.

On the other hand, Cu^1+^ storage is regulated at the hepatic level, mainly by metallothioneins (MTs), which are a family of low-molecular-weight proteins with high cysteine content, allowing them to efficiently bind various metals in the body. In humans, MTs are grouped into four families: MT1, MT2, MT3, and MT4. The MT1 and MT2 isoforms are present in most organs, whereas MT3 is mainly localized in the brain and MT4 in epithelial cells. It is important to highlight that, at the extracellular level, MTs are capable of binding Cu^2+^, which is then reduced to Cu^1+^ through interaction with sulfhydryl (-SH) groups, promoting its reduction [45].

Studies on copper supplementation published to date have reported that factors such as the type of metal salt and particle size are important aspects to consider in its distribution. In vitro studies have suggested that metallo-organic complexes promote an increase in intracellular metal concentration compared to inorganic salts [46]. However, the expression of proteins regulating copper homeostasis is similarly increased by both organic and inorganic salts; in some cases, expression is even higher, depending on the cell line when exposed to an inorganic source, suggesting that copper absorbed from organic salts may utilize alternative internalization mechanisms different from transporter-mediated pathways [46,47] and that this process is further facilitated by the size of the bound organic molecule [47]. Another relevant aspect is that copper derived from organic sources promotes the expression and activity of cuproenzymes such as Zn/Cu SOD while causing a slight decrease in glutathione (GSH), one of the main cellular antioxidant systems [46]. Additionally, evidence from animal models indicates that copper from organic salts shows higher bioavailability and improves evaluated parameters in various studies [48,49,50].

In in vivo studies, Cholewińska [51] demonstrated that copper’s bioavailability in tissues increases with smaller particle sizes and that this delays the time to reach the maximum Cu^2+^ concentration (t_max_). Furthermore, it has been shown that the median lethal dose (LD_50_) is higher when evaluated in nano- or microstructured copper systems compared to ionic salt administration due to changes in physicochemical properties such as electrostatic charge and the particle surface area [52,53].

Although LD_50_ values might suggest lower toxicity for micro- and nanostructures, several studies have shown that metallic nanoparticles can induce the formation of reactive oxygen species (ROS), compromising cellular integrity when enzymatic and non-enzymatic antioxidant systems are overwhelmed [54]. This imbalance promotes oxidative stress and triggers the oxidation of lipids, proteins, and nucleic acids, affecting cell viability and tissue function [55,56]. Furthermore, free metal ions can participate in Fenton-type redox reactions and promote the generation of hydroxyl radicals, so high intracellular copper concentrations are not always beneficial [57]. Even though the wide biodistribution of micro- and nanostructured systems could decrease therapeutic specificity and promote ROS production in different tissues, the aim of reducing particle size is to decrease the required dose and, consequently, the risk of toxicity. Consequently, preclinical studies will be necessary to define the optimal dose, characterize tissue biodistribution, and establish the pharmacokinetic parameters required to design safe chronic administration regimens before proceeding with translational studies. From a technological perspective, the use of ligands with a higher affinity for copper than endogenous ligands could increase the stability of micro- and nanostructured systems; promote controlled release of the metal; and enhance physicochemical modular properties that impact their lipophilicity, biodistribution, cellular uptake, and redox behavior.

To study the biodistribution of exogenous copper in healthy volunteers, copper isotope Cu^64^ has been used. Results demonstrate that intravenous administration promotes accumulation of the metal in the liver, intestine, and pancreas, while a clear first-pass effect is observed after oral administration. Following copper administration, the organism does not rely on rapid elimination but, rather, on a highly regulated system that restores systemic homeostasis. Therefore, in response to increased systemic copper levels, compensatory mechanisms are activated, including reduced intestinal absorption and increased biliary excretion, which is considered the main route of metal elimination [58,59].

Copper intoxication in humans is possible although uncommon in healthy individuals due to the homeostatic mechanisms that regulate its absorption, distribution, and excretion, as previously described. Under physiological conditions, the liver plays a central role in controlling excess copper through biliary excretion, preventing systemic accumulation. Additionally, it is important to consider that the toxicity of micro- and nanosystems based on copper complexes depends on structural solubility, the administered dose, and the duration of exposure. Therefore, during the development of these systems, in vitro and in vivo studies should be included to evaluate their kinetics, biodistribution, and toxicity.

## 3. The Role of Copper Complexes in Therapeutic Strategies

In recent years, the properties of copper metal ions and their reactive behavior in conjunction with the ligands to which they bind have been investigated [60]. These properties (antimicrobial, antifungal, cytotoxic, and enzymatic cofactors) are considered useful for the development of new therapies for different diseases. Unlike simple salts, metal complexes are of particular interest in drug development due to their non-linear geometry and diverse stereochemical properties [14], characteristics that enable them to interact with biomolecules through unconventional mechanisms of action, such as ligand exchange after the complex has been administered [61].

The bioactive properties of these complexes can be exploited in the medical field, for example, for the treatment of different types of tumors, including glioblastoma [62] and cancers of the colon [63], as well as endometrial [64], breast [65] or pancreatic [66] cancers, through their cytotoxic activity. For example, Emami [67] demonstrated that Cu^2+^ complexes with curcumin and 2,2′-bipyridine-5,5′-dicarboxylic acid (BPYD) exhibited cytotoxicity in breast cancer cells (MDA-MB-231) and a lower cytotoxic effect in healthy cells, showing a greater effect than the reference therapy. On the other hand, Cu^1+^ complexes with phosphine and thiosemicarbazone ligands were shown to be effective in a prostate cancer PC3 cell model [68]; meanwhile, Cu^1+^ complexes with redox-active azothioformamides (ATFs) and Cu^2+^ complexes with polyethyleneimine showed efficacy against lymphoblastic leukemia cells (K562) and MCF-7 breast cancer cells, respectively [69,70]. The use of drugs such as disulfiram [64] or amantadine [62] as ligands has also been described. These drugs exhibit a cytotoxic effect on cancer cells, thereby avoiding an effect on healthy tissues. This not only enables the repurposing of these drugs by conferring new therapeutic activity but also demonstrates that copper is an anticancer agent.

**Figure 2:**
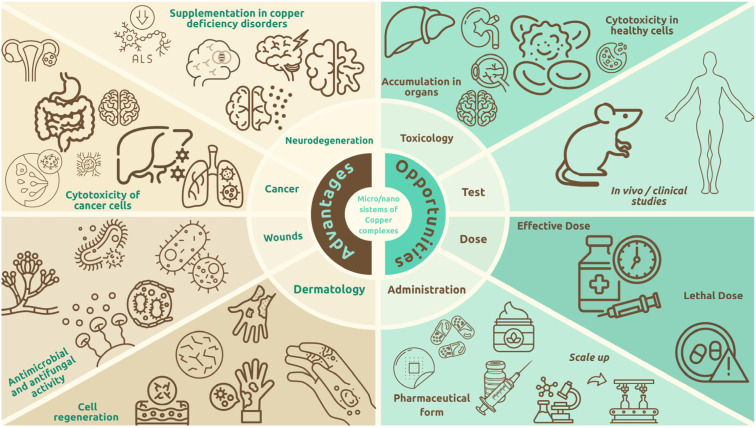
Advances and opportunities in micro- and nanostructured copper-complex systems.

Furthermore, copper complexes with sophorolipids [71] or triazoles [72] have been found to exhibit antifungal, antiparasitic, and antibacterial effects, achieving the elimination of organisms such as *Leishmania amazonensis*. Their use has also been studied in the treatment of infections and wounds [73], promoting the recruitment of macrophages to these areas [33], and in fungal infections such as keratitis through the disruption of biofilms formed by fungi like *Candida albicans* [73] (Figure 2).

In this context, numerous examples of copper complexes have been developed using amino acids, Schiff bases, triazoles, flavonoids, sophorolipids, and phenothrolins as ligands, which have shown potential as anticancer, antimicrobial, antifungal, or antioxidant drugs. Table 1 highlights some of these structures, along with their physicochemical properties and whether in silico, in vitro, in vivo, or toxicity evaluations have been reported.

However, despite the progress that has been made, there are still several areas of opportunity, since most of these compounds are in the stages of synthesis; pharmaceutical development; or, in some cases, the preclinical phase—and only a few reported molecules have been evaluated in in vivo studies [64,72,80,86,87]. Among the complexes described previously (Table 1), only Cu-ATSM and copper histidinate have pharmacokinetic data from patient studies. The determination of C_max_, T_max_, and t_1/2_ allows for the design of safe therapeutic regimens. Furthermore, the high volume of biodistribution (Vd) suggests significant bioaccumulation in organs, as metal elimination is slow.

Therefore, further studies will be necessary for the remaining complexes, as the instability of Cu^2+^ complexes in physiological environments is one of the main barriers to their clinical application. A significant limitation of Cu^2+^ complexes is their variable stability under physiological conditions, which is strongly influenced by both the ligand structure and the biological environment. Martínez-Camarena [88] demonstrated the degradation of the complex at pH 7.4, releasing copper ions. Therefore, dynamic processes such as ligand dissociation, transchelation with serum proteins [44], and solubility-dependent precipitation can substantially alter copper speciation [89] and ultimately affect biodistribution, cellular uptake, and biological activity. 

The release of copper from metal complexes occurs primarily through exchange reactions with endogenous biomolecules and degradation [90]. Its rate is determined by thermodynamic stability, as well as biological microenvironmental factors such as pH and cellular redox state.

A fundamental part of achieving effective therapy with copper complexes is designing suitable pharmaceutical formulations for these types of molecules, since most are unstable in solution. These formulations must facilitate the administration, absorption, permeation, distribution, and elimination of the compounds, ensuring that they reach the target tissues at therapeutic concentrations. Furthermore, careful pharmaceutical design minimizes the release of free copper ions, reducing their systemic toxicity and adverse effects. It also contributes to improved bioavailability, protects complexes from metabolic degradation, prolongs their half-life in the body, and optimizes interaction with specific biomolecules. This design may consider the use of formulations based on micro- and nanostructured systems, which allow for controlled and targeted release of the complexes. Finally, well-designed formulations can allow for localized or controlled administration, which is especially relevant in topical applications to wounds or specific tissues, increasing therapeutic efficacy and reducing unnecessary exposure of another organ.

## 4. Pharmaceutical Innovations in Copper Complexes

A novel drug delivery system must be designed to overcome the critical limitations of conventional release. The fundamental characteristics of these systems focus on their ability to modulate the release of the active pharmaceutical ingredient or ensure targeted delivery to specific tissues. Furthermore, they must exhibit stability to prevent physical, chemical, or enzymatic degradation before reaching the target site. Additionally, biocompatibility is essential to guarantee the absence of responses associated with toxicity, carcinogenicity, reproductive toxicity, or immunogenicity; therefore, approaches must be sought to develop formulations that optimize the appropriate microenvironment for the drug.

In pharmaceutical technology, microparticulate systems include microparticles, microspheres, microcapsules, and microemulsions, each offering distinct advantages for drug delivery applications. The implementation of microparticles allows for the encapsulation of copper complexes within matrices that enhance their stability and enhance their distribution. A significant advancement is the use of “smart” systems that respond to microenvironmental stimuli, such as pH or reactive oxygen species (ROS), thereby enabling programmed release at the site of action [91,92], while the use of biopolymers such as alginate or chitosan derivatives has enabled the creation of biodegradable and biocompatible microparticles that act as multifunctional scaffolds. Ruggeri [93] developed copper-doped biomimetic microparticles and clays for the treatment of chronic wounds and infections. Utilizing the spray-drying technique, they employed chitosan carbamate and montmorillonite doped with Cu^2+^ ions. The advantage of their process lies in the absence of organic solvents, allowing the microparticles to function as multifunctional scaffolds that promote cell proliferation, control bleeding, and modulate copper release. This provided a formulation with antimicrobial activity against pathogens such as *S. aureus* and *E. coli*. Subsequently, Schio [94] developed a film-type formulation featuring copper microparticles using the drop-casting technique, which proved to be reproducible and cost-effective. Implementing microparticles in aqueous systems or hydrogels as an injectable depot enables controlled and targeted release, thereby extending the therapeutic window for administering these compounds. For example, these systems were evaluated against murine hepatitis virus type 3 (MHV-3), used as an analogous model for other coronaviruses. The results suggest that both the micrometric size and the oxidation state of copper are the most important variables.

On the other hand, a study by Strauch [95] compared the potential application of copper nanoparticles versus microparticles and evaluated their effect on oxidative stress and DNA damage in human lung cells. They highlighted that nanoparticles exhibit greater cytotoxicity and genotoxicity due to their greater penetration potential and the intracellular release of copper ions. Furthermore, they emphasized that microparticles, due to their size, have the capacity to modulate oxidative stress by inducing the expression of genes such as heme oxygenase 1 (HMOX1) and activating an antioxidant defense response. Additionally, increases in the expression of metallothioneins (*MT1X* and *MT2A*) and heat-shock response genes such as heat shock protein family A (Hsp70) member 1A (*HSPA1A*) were observed. Another more recently reported microsystem is copper MOF-74, a porous metal–organic framework with high thermal and chemical stability. Its open structure enables the controlled release of Cu^2+^ ions, conferring antimicrobial and therapeutic properties. Furthermore, its large specific surface area and high adsorption capacity make it especially promising for biomedical and pharmacological applications [36]. Table 2 presents some of the characteristics of pharmaceutical formulations (polymeric, non-polymeric, and material synthesis-based) of micro- and nanostructured systems whose objective is the delivery and release of copper.

**Figure 3:**
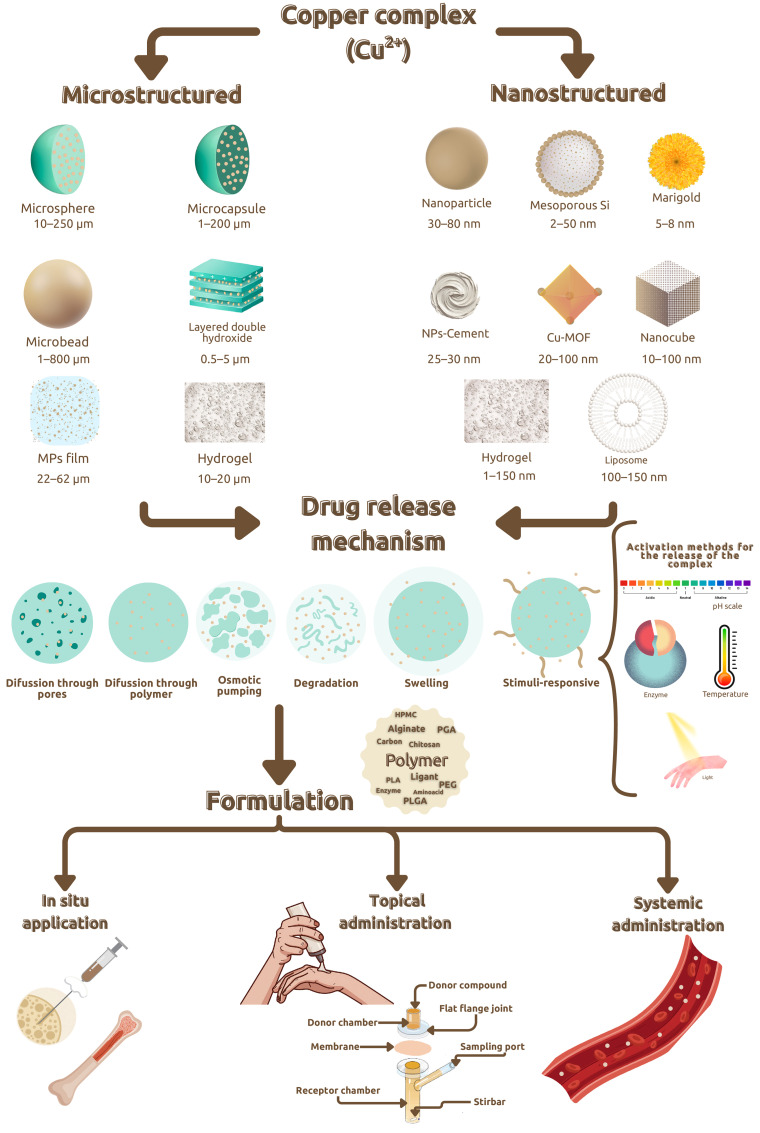
Copper-based micro- and nanostructured systems, their controlled drug-release strategies, and general routes of administration.

The use of copper complexes at the nanoscale has transcended their traditional role as nutritional supplements, evolving into high-precision active therapeutic agents. Current research focuses on overcoming the systemic toxicity of free ions through the design of nanoparticulated systems that increase the bioavailability of the metal in specific tissues [102] through different responses to physiological stimuli generated by the microenvironment of diseases. Figure 3 shows some of the different types of nano- and microstructure systems used for copper delivery, as well as the possible activation mechanisms for ion release.

Copper nanoparticles and their chelates integrated into polydopamine structures have been utilized to enhance photothermal therapy due to their excellent light-to-heat conversion efficiency. Such nanosystems leverage the capability of polydopamine to chelate metal ions and load chemotherapeutic drugs, enabling smart, pH-sensitive release within the tumor microenvironment. Such applications aim to overcome current limitations in glioma treatment, such as low survival rates and drug resistance, while simultaneously improving penetration across the blood–brain barrier [103].

In materials science, reports exist on the development of ultra-small copper nanoparticles synthesized from copper chloride and ascorbic acid. These act as nanozymes by mimicking the activity of catalase, superoxide dismutase, and glutathione peroxidase to eliminate ROS, additionally aiding in wound healing, reducing acute liver inflammation, and exhibiting cytoprotective effects [104]. Yordanova [102] evaluated the biological properties and eco-safety of silica–copper nanoparticles synthesized via the sol–gel method, using tetraethyl orthosilicate and copper hydroxide as precursors. The formulation achieved 100% inhibition of key pathogens through controlled Cu^2+^ release. However, despite showing concentration-dependent selectivity in human cells, its significant acute toxicity in Daphnia magna poses a challenge. These findings underscore the potency of copper nanoparticles as antimicrobial agents while highlighting the need to balance therapeutic efficacy with environmental safety.

As a precedent for in situ formulations, the development of copper-based biomaterials has emerged as a key strategy in regenerative medicine due to their ability to simultaneously stimulate osteogenesis and angiogenesis. The release of Cu^2+^ activates hypoxia-inducible factor 1-alpha (HIF-1α) and vascular endothelial growth factor (VEGF) expression, promoting the formation of new blood vessels essential for bone-graft viability. In parallel, copper enhances the differentiation of mesenchymal stem cells toward the osteoblastic lineage, increasing the activity of enzymes such as alkaline phosphatase and lysyl oxidase, thereby favoring calcium and phosphorus deposition, as well as collagen and keratin synthesis [105]. Furthermore, copper nanocements have been designed as an alternative to prevent infections in bone surgeries and implants. These cements inhibit bacterial growth through the sustained release of Cu^2+^, outperforming cements with gentamicin. However, at high concentrations, they can cause cytotoxicity, decrease osteoblast viability, and cause hemolysis, so it is necessary to optimize the copper load [106].

To address the inherent complexity of nanotechnology-based therapeutics, the FDA has issued dedicated guidance aiming to implement a risk-management framework. This approach evaluates how nanoscale attributes influence the safety and efficacy of drug products. The document outlines recommendations for non-clinical and clinical evaluations, criteria for physicochemical characterization, and quality-control strategies throughout the manufacturing process. Furthermore, while not explicitly mandated, it is highly recommended to complement these assessments with relevant ICH safety guidelines [107,108]. It is also recommended to evaluate pharmacokinetic studies using the general parameters (AUC, C_max_, t_max_, t_1/2_, CL_renal_, CL_total_, and Vd) for free and total drugs, dose-proportional and exposure–response studies, clinical comparison with non-nanoscale products, and release characteristics.

Despite their therapeutic potential, the clinical translation of nanoproducts is often hindered. To provide a comprehensive overview, Table 3 summarizes the distinct advantages, limitations, drug-release characteristics, and scale-up challenges associated with prominent nanotherapeutic products. Critical hurdles include characterization complexities arising from morphological diversity and high polydispersity indexes, unpredictable genotoxic risks, tissue accumulation, environmental safety concerns, and enhanced permeability across biological barriers such as the blood–brain barrier.

Although little has been reported on the limitations of the formulations developed in these studies, several critical attributes must be controlled, such as the particle size distribution, physicochemical stability, encapsulation efficiency, and metal-ion release kinetics during the development of liposomal gels, hydrogels, composites, patches, electrolyzed nanofibers, or MOFs [109]. Consequently, one of the main challenges is developing robust, reproducible, and easily manufactured pharmaceutical platforms that ensure controlled metal release, selective tissue distribution, and a suitable long-term safety profile.

**Table 3:** Comparative analysis of the advantages, disadvantages, release profiles, and manufacturing challenges of copper-based micro- and nanosystems.

System	Advantages	Disadvantages and Limitations	Release Profile	Manufacturing and Scale-Up Challenges	Reference
Liposomes	-High compatibility with the encapsulation of Cu ions in aqueous media -Co-encapsulation of drugs with different polarities -Enhanced targeted drug-delivery capabilities -Biomimetic evasion of the mononuclear phagocyte system	-Unstabilized formulations may induce oxidative stress -Risk of copper overload, hepatic accumulation, and subsequent neurological abnormalities -Low release efficiency (<50% in 50 h) -Use of hazardous and toxic organic solvents during formulation	-Biphasic release kinetics (initial burst effect followed by prolonged release)-Targeted release: -Active targeting (via antibodies, peptides, and folates as ligands) -Temperature-mediated -pH-mediated (gastrointestinal and tumoral) -Light-triggered	-Terminal sterilization by heat or gamma-radiation is not feasible (drug and liposome degradation) -Low reproducibility at large scale -Long manufacturing time and limited availability of specialized equipment for industrial batches -Low encapsulation efficiency and limited long-term stability	[110,111]
MOFs	-High porosity, ensuring high Cu loading capacity -Intrinsic pro-angiogenic activity -Versatile carriers for drug delivery in cancer treatment -Localized delivery of drugs and Cu ions for wound healing and diabetic foot ulcers	-Potential cytotoxicity of constituent organic ligands -Highly toxic for human consumption and environmental pollution -Narrow therapeutic index of Cu ions as a drug -Potential to induce pulmonary toxicity	-Controlled release -Targeted release -pH-responsive, driven by polymer degradation and acidic environments in tumoral microenvironments	-Reduced synthesis yields stemming from extensive washing and purification steps -Reliance on high-vacuum processes and prolonged drying periods for surfactant removal -Limited control over the polydispersity index at the industrial scale -Complexities associated with industrial scale-up	[112,113,114,115]
Hydrogels	-Optimal vehicles for topical and localized administration of drugs -Multifunctional therapeutic profiles (antioxidant, anti-inflammatory, and tissue-regenerative) -High biocompatibility and environmental sustainability -Enhanced localized angiogenesis in cutaneous lesions -Non-specific, resistance-independent antimicrobial mechanisms -Safe, non-toxic microparticles of Cu_2_O (MedCu ^®^) have shown no toxicity with cost-effective formulations	-Restricted primarily to localized cutaneous delivery -Poor mechanical strength and integrity -Susceptibility to premature washing out and rapid drug elimination -High batch-to-batch variability -Cytotoxicity of elevated Cu ion concentrations towards healthy fibroblasts -Burst effect and excessive rate release, which exacerbate oxidative stress and chronic inflammation -Limited efficacy of injectable hydrogels in deep, cavitary wounds	-Sustained release of Cu ions in polyester and polypropylene matrixes -Synergistic coupling with photothermal therapy and near-infrared (NIR) laser irradiation for triggered drug release -Targeted release mediated by enzymatic degradation or pH fluctuations	-Challenging industrial-scale purification of toxic residual monomers -Difficulties in removing endotoxins and antigenic proteins from biopolymers, increasing manufacturing costs -Incompatibility with terminal heat sterilization or standard membrane filtration -Liofilization may be necessary to avoid nanoarchitectural damage or alteration in Cu homogeneity -Physicochemical disparities between copper compounds and excipients, leading to sedimentation, agglomeration, and compromised content uniformity	[116,117,118,119,120]
Polymeric micelles	-Enhanced systemic safety profile via localized chelation at the therapeutic target site -Suitability for integration with photodynamic therapy (PDT) -Optimized pharmacokinetic profiles of encapsulated drugs	-High structural complexity -Burst release, triggering inflammatory responses and systemic toxicity -Suboptimal chemical stability -Low drug-loading capacity relative to alternative nanostructures -Reliance on hazardous organic solvents (THF and DMF) during manufacturing	-Controlled release -Targeted release strategies (e.g., pH-responsive release in tumoral microenvironments and active targeting directed at α_v_ and β_3_ integrins in endothelial cells)	-Low synthesis yield due to the need for several purification steps and low encapsulation efficiency -Elevated synthesis and manufacturing costs -Poor batch-to-batch reproducibility -High risk of cross-contamination with template bleeding procedures -Requirement for high volume of organic solvents -Some drugs can suffer precipitation or crystallization; therefore, lyophilization is necessary	[121,122,123,124,125]

From a pharmaceutical perspective, the route of administration is a critical factor in the development of copper complex-based therapies. For dermatological applications addressed in this review, local or topical administration is generally preferred, as it allows for direct deposition of the active compound at the site of action, minimizing systemic exposure. However, effective topical administration of copper complexes requires the barrier function of the stratum corneum be overcome, which limits the penetration of many hydrophilic or highly charged metal complexes [126]. Therefore, the physicochemical properties of the complex, including molecular size, lipophilicity, coordination stability, and charge, must be carefully optimized to achieve adequate skin permeation and retention in the skin layers. Furthermore, employing formulation strategies using lipid and nanometric systems such as creams, hydrogels, nanoparticles, liposomes, and other delivery systems can further enhance local bioavailability and controlled release [119].

Conversely, transdermal administration for this type of condition aims for partial penetration, meaning crossing of the skin barrier to different layers of the skin without necessarily reaching systemic circulation. In this case, copper complexes must possess sufficient permeability to reach the dermal microcirculation while maintaining their chemical stability during transport. Additional considerations include the rate of absorption, prevention of excessive accumulation in the skin, and the ability to provide predictable systemic exposure should it occur when the complex or system has a high partition coefficient. These properties are evaluated through in vitro/ex vivo permeation studies using Franz diffusion cells with synthetic membranes or pig ear skin, respectively, as skin barrier models [127].

## 5. Copper Complexes as Emerging Treatments for Dermatological Diseases

The pharmacological therapeutic landscape based on copper complexes for treating skin conditions has expanded significantly over the past decade (Table 4), driven by a better understanding of copper homeostasis and its pleiotropic functions within physiology. Copper goes beyond being a structural cofactor in enzymatic reactions, as it is also involved in signaling cascades that dictate inflammation, angiogenesis, extracellular matrix remodeling, and immune defense [128,129]. Therefore, copper is intrinsically involved in the structural and functional integrity of skin as an obligate cofactor for lysyl oxidase (collagen/elastin cross-linking), tyrosinase (melanin biosynthesis), and Cu/Zn-SOD1 (antioxidant defense) [126,130]. Disruption of copper homeostasis has been associated with psoriasis, atopic dermatitis, acne vulgaris, non-melanoma skin cancers and wounds [131]. Table 4 mentions other recent advances in the use of copper complexes as new therapeutic strategies for dermatological diseases and in the treatment of infections.

### 5.1. Advances in Skin Cancer Therapeutics

In melanoma, a type of skin cancer derived from melanocytes and characterized by a high capacity for metastatic dissemination, recent evidence suggests that copper homeostasis may play a relevant role in regulating tumor-cell survival. In this context, the administration of copper compounds induces a microenvironment of copper overload that promotes the intracellular accumulation of Cu^2+^, which is reduced to Cu^1+^ by ferredoxin 1 (FDX1). This event is particularly relevant, as FDX1 not only participates in copper reduction but also in the regulation of protein lipoylation through its interaction with lipoic acid synthase (LIAS). This process promotes the post-translational modification of lysine residues present in essential mitochondrial complexes, such as the pyruvate dehydrogenase complex, the α-ketoglutarate dehydrogenase complex, the branched-chain α-ketoacid dehydrogenase complex, and the glycine cleavage system [150].

Subsequently, Cu^1+^ cations interact with lipoylated proteins involved in the tricarboxylic acid cycle, particularly with dihydrolipoamide acetyltransferase (DLAT), a component of the pyruvate dehydrogenase complex, promoting the formation of disulfide bond-dependent aggregates. In addition, excess Cu^1+^ induces the destabilization of proteins containing iron–sulfur (Fe-S) clusters, triggering proteotoxic stress associated with increased expression of molecular chaperone HSP70, elevated production of reactive oxygen species, and mitochondrial dysfunction. Taken together, these events lead to progressive mitochondrial damage and, ultimately, to copper-mediated cell death, a process known as cuprosis, which represents a promising strategy for limiting the proliferation of tumor cells in this type of cancer [151].

A new copper complex acting as a proteasome inhibitor was studied in a 7,12-dimetilbenz[a]anthracene (DMBA)-induced skin squamous cell carcinoma model, demonstrating significant decreases in both proteasome levels and catalytic activity, resulting from the inhibition of 20S proteasome β5 subunit chymotrypsin-like activity, which was corroborated by molecular docking analyses [146]. Moreover, GHK-Cu^2+^ is an epigenetic regulator that can reset gene misregulation that governs tissue destruction and cancer progression by down-regulating metastasis-associated genes while up-regulating DNA repair processes [126].

Zhang [152] reported copper–cysteine coordination complex (Cu^2+^–Cy) nanoparticles that produce ROS upon activation by X-rays, ultraviolet light, microwaves, and ultrasound. Following exposure to X-ray irradiation at 2.5 Gy, Cu^2+^–Cy nanoparticles induced 84.7% apoptosis/necrosis in B16F10 melanoma cells in vitro, in comparison to 24.1% with just the nanoparticles alone. Importantly, in vivo experiments indicated that Cu^2+^–Cy-mediated X-ray photodynamic therapy not only activated antitumor immunity by promoting CD8^+^ T-cell and NK-cell infiltration and dendritic cell maturation but also inhibited immunosuppressive M2 macrophages. These results make Cu^2+^–Cy nanoparticles a platform enabling radiotherapy, oxidative therapy, and immunotherapy for melanoma.

Along similar lines, the use of disulfiram in therapy has provided one more route to copper-mediated treatment of melanoma. Wang [153] showed that disulfiram/copper complexes (DSF/Cu^2+^), combined with ionizing radiation at 12 Gy, induced strong immunogenic cell death in human MV3 and murine B16F10 melanoma cells. In a syngeneic C57BL/6 mouse model, intratumoral DSF/Cu^2+^ injection followed by irradiation provided significantly greater tumor suppression than either modality alone, along with enhanced CD3^+^, CD4^+^, and CD8^+^ T-lymphocyte tumoral infiltration accompanied by a decrease in the quantity of myeloid-derived suppressor cells. These results suggest that copper complexation with disulfiram may be a solution to radioresistance in melanoma.

The inherent resistance of melanoma to standard cytotoxic chemotherapy regimens has prompted the investigation of regimens that combine copper metallodrugs with existing platinum-based agents. Mariani [154] evaluated the [Cu^2+^ (HPClNOL)Cl]Cl complex against murine melanoma B16-F10 cells, finding that only the copper derivative displayed significant cytotoxicity through ROS generation and apoptosis due to the disruption of mitochondrial membrane potential, thereby opening the gate for Cu^2+^ complexes as potential adjuvants for platinum-based melanoma treatment.

Engineered nanocarriers have been explored as alternative strategies to address the poor aqueous solubility of many copper-based metallodrugs, which limits clinical translation. Vieira [155] synthesized polyhedral oligomeric silsesquioxane (POSS) nanocarriers functionalized with 3-amino-1,2,4-triazole-5-carboxylic acid, to which oxindolimine–Cu^2+^ complexes were anchored. POSS nanocarriers markedly increased cytotoxic potency against NRAS-mutated SK-MEL-147 melanoma cells with selectivity compared to non-tumorigenic fibroblasts and facilitated oxidative melanin degradation that was able to directly target a major contributor of chemoresistance in pigmented melanomas. To support the use of copper complexes for this condition, other examples are mentioned in Table 3.

An important consideration for the clinical translation of copper-based therapies in melanoma is patient stratification. Melanoma exhibits metabolic heterogeneity, and susceptibility to copper-mediated cytotoxicity or cuprosis can vary depending on the metabolic status and mitochondrial dependence of each tumor. Therefore, molecular profiling approaches could improve the identification of patients most likely to benefit from these therapies. Potential predictive biomarkers include the expression of genes involved in copper homeostasis and the regulation of cuprosis, such as FDX1, LIAS, DLAT, SLC31A1 (CTR1), ATP7A, and ATP7B, as well as markers associated with activity of the tricarboxylic acid cycle and mitochondrial metabolism. Furthermore, genetic alterations frequently observed in melanoma, including mutations in BRAF, NRAS, and NF1, can influence tumor metabolism and consequently modulate the response to copper-based interventions. While these biomarkers have not yet been clinically validated, integrating molecular and genetic profiling into future studies could facilitate precision medicine approaches and optimize patient selection before the implementation of copper-based therapies in the clinical setting. It is worth noting that several studies suggest that physiological levels of copper promote tumor progression by supporting angiogenesis, proliferation, and redox homeostasis, whereas therapeutic strategies exploit copper dysregulation or overload to induce cytotoxicity, oxidative stress, and cell death [151]. These processes are context-dependent and involve distinct mechanisms, with their effects influenced by copper concentration, subcellular compartmentalization, and the metabolic state of the tumor.

### 5.2. Cellular and Tissue Regeneration Approaches in Wounds and Inflammatory Skin Disorders

**Figure 4:**
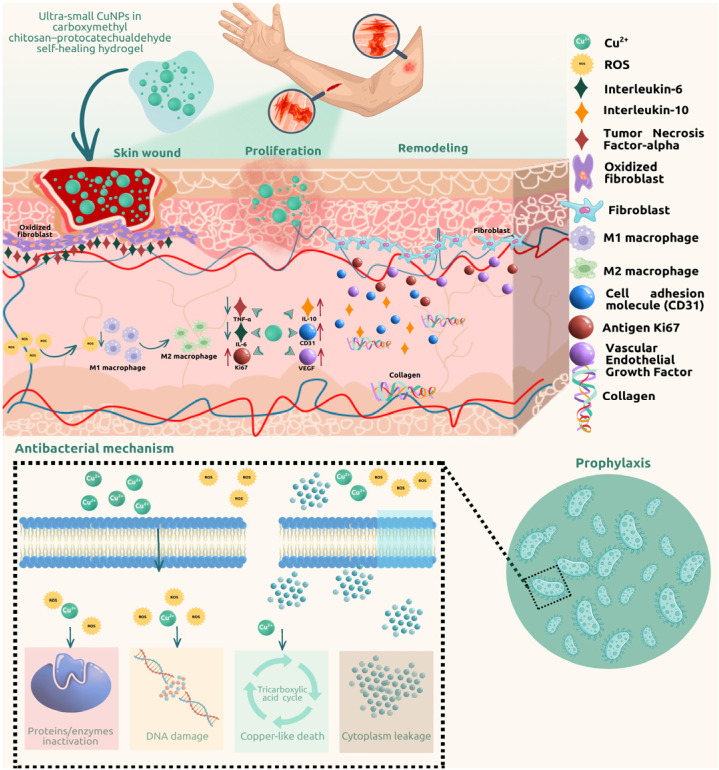
Micro- and nanostructured copper systems in skin diseases.

In the case of cutaneous wounds, a microenvironment corresponding to the inflammatory phase is generated, in which M1-type macrophages predominate. These are associated with the production of ROS and the release of pro-inflammatory cytokines such as tumor necrosis factor (TNF-α) and interleukin-6 (IL-6), which contribute to the elimination of pathogens and cellular debris. Subsequently, the administration of copper complexes at the wound site promotes the transition to M2-type macrophages, stimulating the production of the Ki-67 antigen, CD31 (adhesion molecule), and vascular endothelial growth factor (VEGF). In addition, it reduces the levels of TNF-α and IL-6, thereby promoting angiogenesis, as well as the migration and activation of fibroblasts, and the synthesis and deposition of collagen [156]. Likewise, simultaneous prophylaxis at the wound site is required; therefore, these copper complexes can exert an antibacterial effect, as Cu^2+^ ions induce lipid peroxidation of the bacterial membrane, increase its permeability, and promote the intracellular uptake of the metal. This leads to protein inactivation and the generation of reactive oxygen species (ROS), as well as DNA damage through interactions with sulfur- and phosphate-containing groups. In addition, ATP production is inhibited, and intracellular material is displaced [157] (Figure 4).

Glycyl-L-histidyl-L-lysine (GHK-Cu^2+^) is the most well-studied tripeptide for dermatological purposes. In humans, GHK-Cu^2+^ is found in plasma at concentrations peaking around 200 ng/mL at age 20 and declining to 80 ng/mL by age 60, modulating over 30% of the human genome at biologically relevant doses [126]. The synergism of GHK-Cu^2+^ with hyaluronic acid (HA) increases collagen type IV production by 25.4-fold in dermal fibroblasts and by 2.03-fold in skin explants, implying that the amount of glycosaminoglycan and chondroitin sulfate produced may increase through this mechanism [158]. Recent evidence has also expanded GHK-Cu^2+^ derivatives into cutaneous pigmentation modulation.

Hong [159] developed CP-AcT, combining palmitoyl-GHK-Cu^2+^ with N-acetyl-L-tyrosine. CP-AcT markedly potentiated tyrosinase activity and melanin content in human A375 and murine B16 melanoma cell lines at a non-cytotoxic range (up to 8 µg/mL), as well in HaCaT keratinocytes and human foreskin fibroblasts. The implications of these results suggest potential therapeutic applications for hypopigmentary disorders such as vitiligo. Nanocarrier-based delivery systems of copper complexes have demonstrated preclinical efficacy in the healing of diabetic wounds. Hu [160] demonstrated that 3D-printed dermal scaffolds incorporating copper–epigallocatechin gallate mitigated diabetic wound scarring through simultaneous antibacterial activity and collagen remodeling [119,133].

On the other hand, Podgórska [131] summarized data on trace-element changes in psoriasis, pemphigus vulgaris, atopic dermatitis, acne vulgaris, and seborrheic dermatitis, noting a uniformly elevated serum copper level among subjects with psoriasis. This could represent compensation for an anti-inflammatory response driven by copper-induced oxidative stress, highlighting the efficacy of copper-based therapeutic modalities in modulating the immune-inflammatory axis in skin morbidities [161]. In addition, in normal human dermal fibroblasts treated with tumor necrosis factor alpha (TNF-α), GHK-Cu^2+^ and related copper complexes reduced Interleukin-6 (IL-6) secretion, further suggesting direct modulation of pro-inflammatory cytokine cascades relevant to multiple dermatological conditions [126].

Finally, the damage caused by burns can lead to significant local or systemic consequences [162,163]. This is because burns induce protein denaturation, metabolic alterations, and cell death [164]. Following a burn, multiple response mechanisms are activated to restore homeostasis, ranging from increases in cytokines and inflammatory mediators to physiological changes involving the respiratory, cardiovascular, immune, and nervous systems [165,166,167].

Among the primary mechanisms targeted for promotion following a burn injury are angiogenesis, collagen synthesis, and local re-epithelialization. In vitro studies have demonstrated that copper possesses the capacity to promote the expression of HIF-1α, VEGF, and PECAM-1, all of which are genes involved in the angiogenic process [147]. Furthermore, extensive burns can lead to a significant loss of body-fluid volume and increased vascular permeability, resulting in plasma extravasation. Research has shown that copper can stimulate the expression and activity of matrix metalloproteinases, as well as fibroblast proliferation [148,149,168], which are collectively responsible for the degradation and remodeling of the extracellular matrix, including collagen, elastin, and various proteoglycans [169].

Clinically, patients with severe burns (≥25% total body surface area) exhibit decreases in copper concentrations during the first day of hospitalization [170]. Patients may also exhibit hypocupremia several days post injury [171]. A recent study demonstrated that higher copper concentrations are associated with a better prognosis [172], suggesting that supplementation and monitoring of this micronutrient could promote faster and improved recovery. The last 4 rows of Table 3 show examples of copper complexes used for burns.

### 5.3. Antimicrobial and Antifungal Strategies for Infectious Disease Control

Copper is of particular interest as an alternative antimicrobial agent due to its multimodal mechanisms of action, which substantially reduce the potential for resistance development compared to traditional single-target antibiotics [173].

Copper has been described as possessing antibacterial activity via four linkable mechanisms: (1) disruption of the membrane through electrostatic interactions with phospholipid head groups to induce depolarization and lysis [174]; (2) generation of Fenton-type ROS by Cu^+^/Cu^2+^ redox cycling that generate oxidative damage on lipids, proteins, and nucleic acids [175,176]; (3) mis-metalation of essential microbial enzymes by competitively binding thiol and imidazole groups, which displaces native metal cofactors and disrupts iron–sulfur cluster-dependent bacterial respiration [177]; and, finally, (4) direct DNA interaction, causing strand breaks and replication inhibition [178,179].

On the other hand, Beeton [180] observed that Cu^2+^ complexes with functionalized phenanthroline ligands were significantly active against MRSA biofilms, surpassing vancomycin in biofilm removal assays. Chung [181] showed that Cu^2+^ Schiff base complexes are highly active against methicillin-susceptible and resistant *S. aureus* strains, with MICs (minimum inhibitory concentrations) frequently below those of standard antibiotics. More recently, shikonin–copper coordination nanoparticles exhibited synergistic antibacterial and antibiofilm activities against *S. aureus* via ROS-mediated membrane disruption [182].

The rising rates of invasive fungal infections and increasing resistance to azoles and echinocandins have heightened the search for new antifungal strategies. The coordination of bioactive ligands with Cu^2+^ consistently potentiates antifungal activity, likely due to higher lipophilicity, improved cell permeability, and intracellular ROS formation [183,184]. For instance, copper complexes based on the derivatives of ethylenediamine with chiral terpenes have exhibited significantly higher antifungal activity against *C. albicans*, *Sporobolomyces salmonicolor*, and *Penicillium notatum* in comparison with amphotericin B while simultaneously displaying strong activity against ciprofloxacin-resistant MRSA [185].

Nanotechnology-based approaches have provided additional innovations for copper delivery against the fungus. Azadi [186] developed Fe_3_O_4_@SiO_2_/Schiff-base/Cu^2+^ magnetic nanoparticles that exhibited effective antifungal activity against drug-resistant Candida species with reasonable cytotoxicity, offering magnetic recoverability for potential applications in medical devices. Liu [187] characterized chitosan oligosaccharide–pyridine Schiff base copper complexes acting as slow-release antifungal agents with prolonged action against plant pathogenic fungi, a delivery concept applicable to dermatological and mucosal formulations.

Despite promising results, most of the reported data derive from in vitro studies, and comprehensive in vivo pharmacokinetics, pharmacodynamics, and toxicological assessments remain limited. The therapeutic window requires careful delineation, as host-cell cytotoxicity may occur at high copper concentrations through the same oxidative mechanisms that underlie antimicrobial activity [128,188]. Moreover, sub-inhibitory copper concentrations may paradoxically enhance horizontal gene transfer of resistance determinants, which highlights the importance of accurate dosing and formulation [189]. Antimicrobial activity and the stimulation of tissue regeneration represent essential strategies in the prophylactic management of burn injuries. In this context, copper complexes emerge as a promising therapeutic alternative due to their dual bioactive properties. Although ions applied topically may diffuse into the tissue microenvironment due to the loss of the skin barrier, their behavior is mainly local, as their mobility is restricted to the exudate, the extracellular matrix, and the superficial layers of the tissue [190]. Therefore, permeation into other tissues or organs through systemic circulation is generally limited and depends on factors such as ion concentration, exposure time, the degree of vascularization and inflammation of the wound, the physicochemical properties of the complex, and the route of administration [191]. In this regard, the use of appropriate modified release systems such as hydrogels, dressings, micro- or nanoparticles, or MOF-type structures can promote greater retention in the dermal tissue and minimal release into systemic circulation. For this reason, the evaluation and correlation of in vitro and in vivo studies for each system and copper complex is essential in order to demonstrate that excess copper can be properly regulated by physiological transport and excretion mechanisms [190,192,193].

## 6. Current Landscape and Future Outlook

Copper is primarily obtained through food and dietary supplements [194,195]. However, according to the Food and Drug Administration (FDA) database, there is currently only one approved pharmaceutical product containing copper (II) (Cu^2+^-histidinate), in addition to a copper 64 (Cu^64^) injectable used for tumor diagnosis [196]; neither of these is approved for dermatological treatments.

In this context, copper histidinate, an injectable solution approved by the FDA in 2026 (ClinicalTrials.gov identifier: NCT04074512) [197], was developed and approved exclusively for the treatment of Menkes disease, a disorder characterized by copper deficiency and dysfunction of copper-dependent enzymes. Its development required a comprehensive characterization of the metal complex, including stability studies, evaluation of pharmacokinetic and toxicological parameters; determination of the therapeutic range; and the design of a suitable liquid formulation with excipients intended to improve stability against degradation, promote solubility, and optimize the compound’s bioavailability. This subcutaneous injectable formulation contains a copper–histidinate complex obtained by reacting copper chloride with L-histidine and adjusted to a pH of 7.35 [198].

As background, Guzmán [23] initially demonstrated stability problems in formulations with a stoichiometric ratio of 1:2. Therefore, efforts were focused on improving the complex’s stability. In this regard, it has been reported that modifying the ratio to 1:3 improved the formulation’s stability and shelf life by increasing the complex’s chelating capacity. Furthermore, it was noted that the formulation’s sterility contributed to its improved safety and clinical efficacy.

Currently, copper compounds available for administration, including copper gluconate, copper bisglycinate, and copper citrate [199], are marketed primarily as dietary supplements [200]. Currently, copper compounds available for administration are marketed primarily as dietary supplements, including copper gluconate, copper bisglycinate, and copper citrate [200]. Consequently, it is necessary for the other molecules to be evaluated through preclinical and clinical studies to determine their pharmacokinetic parameters, toxicological profiles, and potential adverse reactions. This information will be crucial for the subsequent development of copper-based drugs that comply with good manufacturing practices and relevant regulatory requirements. A prime example is the ATSM complex, which is currently among the most widely studied and reported copper complexes in the scientific literature.

The FDA is expected to approve a growing number of copper compounds currently undergoing clinical evaluation as therapeutic agents. These include copper sodium chlorophyllin, which was subjected to a phase I clinical trial to assess its safety and immunomodulatory efficacy [201]. Similarly, Cu^64^-ATSM has been evaluated in preclinical studies as a prognostic factor in the treatment of head and neck cancer [202]. Furthermore, the available scientific literature on copper-based micro- and nanosystems for dermatological applications is limited. Among the few reported precedents, a topical liposomal copper dispersion evaluated in a pilot trial in patients with mild to moderate acne stands out [132], which demonstrates a window of opportunity in the field of pharmaceutical technology.

In general, based on this background, once the clinical-trial phase is complete, the challenge of moving towards potential use as a pharmaceutical formulation arises. This involves designing and obtaining the pharmaceutical form from the preformulation stage of each copper complex, as well as establishing and controlling the product’s quality attributes during its development. However, after technology transfer and eventual large-scale production, it will be necessary to define and control aspects related to the complex’s stability, the involved unit operations, and the design space in order to guarantee patient access to these copper complexes.

The clinical application of micro- or nanoparticulate systems for controlled drug delivery still faces significant challenges related to their production. Many of the methods used in research, such as solvent evaporation emulsification, nanoprecipitation, and spray drying, allow for precise control of critical variables during small-scale synthesis [203]. However, scaling up production can induce variations in key parameters such as particle size, polydispersity, encapsulation efficiency, and system stability. These variations are usually attributed to changes in mixing dynamics, mass transfer, and the control of the physicochemical conditions of the process.

Furthermore, the inherent complexity of some polymer-based systems used to stabilize copper components has highlighted that maintaining batch-to-batch reproducibility and ensuring critical quality attributes represents a significant challenge for pharmaceutical development [204]. In this context, several studies emphasize the need to design manufacturing strategies and process controls from the earliest formulation stages, thereby facilitating the transition to robust and reproducible production processes. One example of this is the use of microfluidic systems and micromixers [205], which allow for precise control of synthesis conditions and improve the scalability and reproducibility of copper complex-based formulation processes.

### Knowledge Gaps and Research Directions

Despite the breadth of preclinical evidence summarized in this review, several knowledge gaps must be addressed before copper-based micro- and nanosystems can be reliably translated into clinical practice. A recurring limitation is the absence of systematic dose-optimization studies. Because copper operates within a narrow therapeutic window in which the concentrations required for antimicrobial, pro-angiogenic, or cytotoxic (cuproptotic) activity may overlap with those that trigger oxidative damage to host tissues, the definition of an effective yet safe dose for each indication remains largely empirical [128,188]. Future work should prioritize formal dose–response characterization, ideally coupling stimulus-responsive release (pH- or ROS-triggered) with the local copper requirement of the target tissue to maximize therapeutic exposure while minimizing systemic spillover [91,92].

A second gap concerns biodistribution and pharmacokinetics. Most studies report biological activity without quantifying the absorption, tissue distribution, organ accumulation, or elimination of the copper payload. For topical and wound-targeted systems, standardized in vitro release and permeation assays should be correlated with in vivo biodistribution to confirm that copper remains predominantly local and that any fraction reaching the systemic circulation is handled by physiological transport and excretion pathways [190,191,192,193]. For systemically administered complexes, in vivo pharmacokinetic and biodistribution data are indispensable for anticipating hepatic and renal exposure and establishing adequate safety margins.

Third, the long-term consequences of repeated or sustained copper delivery with respect to systemic copper homeostasis remain poorly defined. The induction of metallothioneins and antioxidant-response genes is frequently interpreted as evidence of restored homeostasis; however, these responses primarily reflect an adaptive reaction to cellular stress and do not, by themselves, demonstrate a sustained re-equilibration of copper balance in vivo [95]. Therefore, chronic dosing schemes will require longitudinal monitoring of copper-status biomarkers (e.g., serum copper, ceruloplasmin activity, and metallothionein expression) to detect progressive accumulation, particularly for nanostructured carriers that may prolong tissue residence time. Disentangling transient adaptive responses from genuine homeostatic restoration is essential to avoid mistaking a stress signature for a therapeutic endpoint [9].

Fourth, the environmental dimension of copper nanomaterials warrants explicit consideration. The same physicochemical reactivity that confers antimicrobial potency also drives ecotoxicity: copper-based nanoparticles released into aquatic compartments induce oxidative stress, bioaccumulate along the food chain, and have shown acute toxicity in sentinel organisms such as Daphnia magna and early life stages of fish [206]. Eco-safe design principles, including the use of biodegradable matrices, controlled-dissolution chemistries, and life-cycle assessment, should be incorporated early in formulation development rather than being treated as a post hoc regulatory hurdle.

Finally, clinical translation is constrained by the shortage of copper-based active pharmaceutical ingredients approved by regulatory agencies, the limited number of completed clinical trials, and persistent manufacturing challenges related to batch-to-batch reproducibility and the scale-up of micro- and nanostructured systems [203,204,205]. For oncological applications in particular, the heterogeneity of tumor copper metabolism argues for the use of predictive biomarkers such as the expression of copper transporters (CTR1 and ATP7A/B) and cuproptosis effectors (FDX1 and LIAS) to guide patient stratification and to distinguish the physiological role of copper in tumor progression from its therapeutic induction of cytotoxicity [150,151]. Addressing these gaps in an integrated manner, from dose definition and biodistribution to environmental safety, long-term homeostasis, and biomarker-guided patient selection, would allow the field to evolve from a catalog of promising preclinical observations toward a rationally prioritized clinical development pipeline.

## 7. Conclusions

Copper-based pharmacotherapy represents a promising and versatile approach for the treatment multiple conditions associated with copper imbalance and tissue damage. Bioactive copper complexes demonstrate improved bioavailability, favorable distribution in tissues and the brain, reduced toxicity, and a reduced tendency for hepatic accumulation. Advances in pharmaceutical innovation, particularly copper-based micro- and nanosystems, have improved the efficiency and stability of their delivery, supporting their development as novel therapeutic strategies. Evidence highlights their beneficial effects in dermatological conditions, ranging from infection control to the promotion of cell regeneration in burns. Furthermore, these complexes show potential to address copper deficiency associated with neurodegenerative disorders. While initial clinical studies are promising, further well-designed clinical trials are needed to confirm their safety, efficacy, and translational applicability.

## Figures and Tables

**Table 1:** Copper complexes in therapeutic applications.

Copper Complex	Ligand	Physicochemical Properties	Application	Mechanism	Evaluated Concentration and Pharmacokinetics	Studies				Reference
						In Silico	In Vitro	In Vivo	Toxicity	
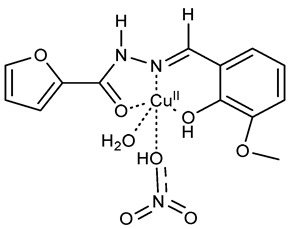 Cu^2+^HL^1^	(E)-N′-(2-hydroxy-3-methoxybenzylidene)furan-2-carbohydrazide (H_2_L^1^) Furan Acylhydrazone derivative	Solid crystal system: Orthorhombic Temperature: 295 (K) Density: 1.398 (mg/m^3^)	Breast cancer	Apoptosis due to the generation of reactive oxygen species (ROS)	IC_50_ 0.25–1 µM	✔	✔	-	-	[74,75]
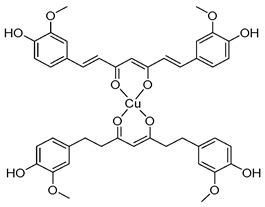 Cu^2+^-Cur	Curcumin	Solid crystal structure Melting point: 137–145 °C Log P: 2.08		Increased expression of adrenal ferredoxin protein (FDX1) and promotion of dihydrolipoamide S-acetyltransferase (DLAT) oligomerization.	IC_50_ 4T-1: 4.77 μM IC_50_ MDA-MB-231: 4.99 μM	-	✔	✔	✔	[76]
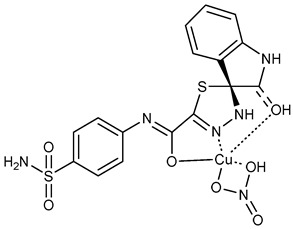 Cu^2+^-[Ltos]NO_3_	Thiadiazole-carboxamide derivative (HLtos)	Melting point: 300 °C > Polarity: Low	Antibacterial and antifungal	Inhibition of respiratory and enzymatic processes; oxidative stress	MIC for bacterial growth: 1.75–2.25 μM MIC for fungal growth: 1.5–1.75 μM	✔	✔	-	✔	[77]
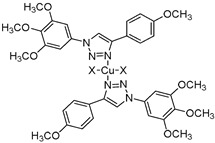 Complex Cl Complex Br	1,4-Disubstituted-1,2,3-Triazole	White solid Melting point: 155 °C	Antiparasitic	ROS generation induces apoptosis in promastigotes	IC_50_ Cl: 0.4 μM IC_50_ Br: 12 μM	-	✔	✔	✔	[72,78]
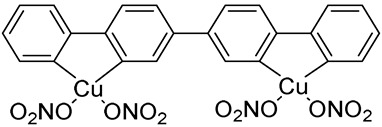 Tetrapyridine-copper complex (B2)	2,2′:5′,5″:2″,2‴-Tetrapyridine	Dinuclear complex Z-potential: DMSO (3.27 mV) and water (4.10 mV)	Antiviral	The coordination of copper in tetrapyridine improved its affinity for the 3CLpro enzyme binding site	IC_50_ B2: 14.03 μM SI BEAS-2B: 2.01 SI MCF-7: 1.35 SI A549: 2.8	✔	✔	-	✔	[79]
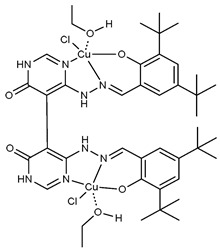 C1	4-Hydroxy-6-hydrazinylpyrimidine (L1)	Binuclear complex Density:1.291 g/cm ^3^	Cancer: lung, stomach and breast	Induction of cuproptosis; generation of mitochondrial superoxide; intracellular ROS; DNA damage; activation of apoptosis	IC_50_ A549: 9.34 μM IC_50_ MGC-803: 3.02 μM IC_50_ MCF-7: 7.37 μM	-	✔	✔	✔	[80]
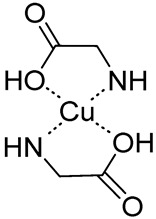 Copper Bis-Glycinate	Glycine	Crystalline solid High water solubility Melting point: approximately 150–200 °C LogP ~ −1 to 0	Immune cell modulating effect	ROS scavenging and anti-inflammatory immunomodulation through reduction in pro-inflammatory cytokine secretion and inhibition of intracellular Ca^2+^ influx in activated immune cells	0.05–100 μg/mL	-	✔	-	✔	[81]
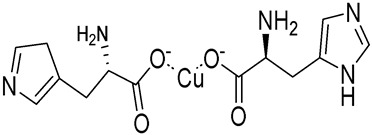 Copper histidinate	Histidine	Crystalline solid High water solubility Melting point: around 200–210 °C LogP ~ −1 to 0	Menkes disease	Cu^2+^ is released from histidine and used as an essential cofactor in multiple enzymes: cytochrome c oxidase, superoxide dismutase (SOD), lysyl oxidase, and dopamine β-hydroxylase.	250 μM/dose (0.5 mL) every 24 h. C_max_: 67 ng/mL T_max_: 0.75 h T_1/2_: 75 h Vd: 1034 ± (588) L AUC_totalx_: 296 ng·h/mL	✔	✔	✔	-	[22,23,82]
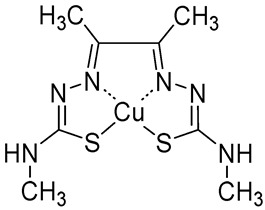 Cu^2+^-ATSM	Diacetyl-bis(N4-methylthiosemicarbazone (ATSM)	Crystalline solid or powder Moderately soluble in water Soluble in DMSO and ethanol	Grade 4 high-grade gliomas	Redox-dependent Cu^+^ transfer to SOD1 intracellular reduction of Cu^2+^(ATSM); retention under Hypoxia, leading to oxidative DNA damage, γH2AX induction, double-strand breaks, and apoptosis.	DMT: 3.7 MBq C_max_: 6.80 ng/mL T_max_: 3.33 × 10^−2^ h T_1/2_: 3.58 × 10^−2^ h AUC_total_: 4.61 ng·h/mL	-	✔	✔	✔	[83,84,85]

**Table 2:** Copper-based micro- and nanostructured systems for therapeutic applications.

	System	Size	Main Component	Release Behavior	Function	Reference
Microestructured	Thermosensitive hydrogel system (Cu^2+^)	10–20 µm	PLGA	Long-term	Anti-tumor	[96]
	Composite films (Cu^1+^)	CuMPs 22–45 µm CuNPs 24–60 µm Cu_2_OMPs 42–62 µm	PVA	Photo-responsive drug delivery	Virucidal	[94]
	Smart nanocomposite- based biomimetic microparticles (Cu^2+^)	32–42 µm	Chitosan	Long-term	Cicatrization Antimicrobial	[93]
	Microparticles chelated with Cu^2+^ ions	2.95 µm	PAS	Non-releasable/catalytic activity (nanozyme)	Antioxidant activity	[97]
	Ion-sensitive hydrogel	-	DGG	Prolongs drug release	Cytotoxicity	[98]
Nanoestructured	PEGylated liposomes incorporating copper oleate into the lipid bilayer (Cu^2+^)	109.6 nm	Lipoid E80 and DSPE-PEG2000	Biphasic (initial burst/prolonged)	Anti-tumor	[99]
	Nanoliposome	135.6 ± 1.8 nm	Poly(vinylpyrrolidone)	Non-releasable/transporter/bioavailability enhancer		[100]
	Micellar nanocarriers (Cu^2+^)	23–26 nm	Pluronic^®^ F127	Achieve targeted drug		[101]
	Nanomaterials/hybrid composites Silica (Si)–copper (Cu^2+^)	25–30 nm	Tetraethyl orthosilane (TEOS)	Non-releasable/ Cu immobilized	Antibacterial Antioxidant activity	[102]

**Table 4:** Effect of copper complexes on dermatological conditions.

	Copper Compound/Complex	Study Type	Disease	Mechanism of Action	Main Limitations	Reference
Formulation	Sodium copper chlorophyllin complex (0.1% liposomal gel)	Clinical trial (pilot)	Acne vulgaris	Antioxidant and anti-inflammatory activity of copper chlorophyllin; wound-healing promotion	- Small, uncontrolled pilot (*n* = 10) - No pharmacokinetic or controlled efficacy data	[132]
	Cunps@CMCS-PCA hydrogel (ultra-small Cu^2+^NPs in carboxymethyl chitosan–protocatechualdehyde self-healing hydrogel)	In vitro In vivo	Diabetic wound healing	Angiogenesis via ATP7A-mediated autophagy inhibition (VEGF/VEGFR2 upregulation); anti-inflammatory via JAK2/STAT3 pathway (M1 → M2 macrophage polarization); antibacterial; self-healing hydrogel scaffold	- Rodent models only - Biodistribution and long-term local copper retention undefined - No clinical validation	[119]
	PLA/Ch- Cu^2+^NPs (copper nanoparticle-loaded polylactic acid/chitosan electrospun patches)		Infected wound healing	Broad-spectrum antibacterial via Cu^2+^ ion release; reduced exudative and inflammatory responses; promotion of tissue regeneration; immunomodulation (CD68+, CD163+, and MPO+)	- Absence of permeation/PK data - Potential local cytotoxicity - Reproducibility and scale-up of electrospun systems unproven	[133]
	M- Cu^2+^@Fh (MHC-I membrane-encapsulated Cu^2+^@ferrihydrite nanoparticles)		Melanoma	Blue light-induced ferroptosis + cuproptosis; MHC-I upregulation via the NF-κB-SUSD6 axis; DC maturation; CD8^+^/CD4^+^ T-cell activation; cold-to-hot tumor conversion	- Heterogeneity of tumor copper metabolism and lack of patient stratification (CTR1, ATP7A/B, and FDX1) - Narrow therapeutic window	[134]
Carrier/system	CA/Lig//N–vanillidene–phenylthiazole Cu^2+^ complex (electrospun tri-component nanofibers)	In vitro	Diaper dermatitis	Antibacterial activity via Cu^2+^-complex release from nanofibers; induction of protein leakage; improved wettability and absorbency via lignin incorporation	- In vitro only - Skin tolerability, permeation, and in vivo efficacy not assessed	[135]
	Copper nanoparticles (Cu^2+^NPs; 40 nm and 80 nm sizes)	In vitro In vivo	Wound healing (acute full-thickness defect)	Enhanced endothelial-cell migration and proliferation; neovascularization; formation of granulation tissue; no hepatic accumulation	- Size-dependent cytotoxicity/genotoxicity of NPs - No PK or long-term safety data - Preclinical only	[136]
	Cu^2+^-EGCG (copper–epigallocatechin gallate metal–polyphenol nanocomposite)		Tumors/drug-resistant wounds infections	Chemodynamic therapy (Fenton-like reaction: Cu^+^ + H_2_O_2_ → ·OH); photothermal therapy (808 nm NIR photothermal); photodynamic therapy (singlet oxygen ^1^O_2_); GSH depletion by Cu^2+^; confirmed by Density Functional Theory calculations	- Rodent models only - Biodistribution and long-term local copper retention undefined - No clinical validation	[137]
	Cu^2+^ (N-N)(bzac)(X) series (N-N = phen/bpy; Hbzac = 1-phenyl-1,3-butanedione)	In Silico In vitro	Melanoma	ROS scavenging (O_2_^−^•, HO• by EPR); ADME-Tox: drug-likeness and affinity for TDP1/KLF5 targets	- Heterogeneity of tumor copper-metabolism and lack of patient stratification (CTR1, ATP7A/B, and FDX1) - Narrow therapeutic window - Off-target toxicity - No clinical translation	[138]
	Cu^2+^ (R-Im)_2_(Macr)_2_ series (R-Im = substituted imidazoles; Macr = methacrylate)	In vitro		Micromolar-range cytotoxicity; distorted octahedral/square-pyramidal Cu^2+^ geometry; H-bond supramolecular networks		[139]
	[Cu^2+^ (bpy)_2_(μ_2_OClO_3_)]ClO_4_ and [Cu(phen)_2_(OH_2_)](ClO_4_)_2_			DNA intercalation; ROS generation; selective cytotoxicity		[140]
	DS- Cu^2+^@PVA (disulfiram– Cu^2+^ in poly(vinyl alcohol) electrospun nanofibers)			ROS generation; GSH depletion; caspase 3 activation; p53/p21 upregulation; BCL-2/β-catenin/cyclin D downregulation; apoptosis (intrinsic pathway)		[141]
	Cu^2+^–thiosemicarbazone complexes (CuT1, CuT10, CuT12, CuT13, and CuT16)			DNA damage (AP sites); G2/M cell-cycle arrest; apoptosis; dysregulation of antioxidant enzyme expression; oxidative stress response; gene alterations		[142]
	Cu^1+^BTC@DDTC MOF (DDTC- Cu^1+^ on Cu^1+^-BTC metal–organic framework)	In vitro In vivo		SLC7A11/GPX4-mediated ferroptosis; proteasome inhibition via DDTC- Cu^1+^; synergism with low-dose cisplatin		[143]
	ZSIDH (ZGGO@SS@Cu/ZIF-8-ICG:DC_AC50@HA)—long-afterglow core-shell NPs (<200 nm)			Cuproptosis; GSH depletion; ICG-mediated photothermal effect; NIR long-afterglow bioimaging; HA-targeted delivery		[144]
	Cu^2+^-based Schiff base complex			Apoptosis induction; G0/G1 cell-cycle arrest; inhibition of intra-tumoral angiogenesis (reduced CD34, VEGF, and bFGF); TUNEL-positive apoptotic cells		[145]
	Novel Cu^2+^complex (structure not detailed in abstract)	In vivo		Proteasome inhibition in tumor tissue		[146]
	CuCl_2_	In vitro	Environmental conditions of hypoxia	Angiogenesis; gene expression of β-actin, VEGF, HIF-1α, and PECAM-1; ability to form tubular structures; cell cytotoxicity	- Compromised barrier widens systemic-exposure risk - No in vivo PK or systemic copper monitoring	[147]
	Cu^2+^ as cupric sulfate in combination with amino acids (L-proline, L-alinine, L-valine, L-leucine, and L-lysine)			Cell viability; expression of collagen and elastin proteins; gene expression of *COL1A2* and *ELN*	- Surrogate molecular endpoints - Few controlled clinical studies	[148]
	Copper–polydopamine Janus nanoparticles (CuCl_2_ as the source of copper)	In vitro/In vivo	Wound-healing model	Antibacterial activity, fibroblasts viability, and collagen synthesis	- Absence of permeation/PK data - Potential local cytotoxicity	[149]
	Cuprous oxide or copper iodide	Ex vivo	Soldering iron	Anti-inflammatory activity (reduced IL-6, IL8, and TGF-β) and keratinocyte proliferation	- Ex vivo organ culture - Compromised barrier widens systemic-exposure risk - No in vivo PK or systemic copper monitoring	[31]

Abbreviations: CMCS–PCA, carboxymethyl chitosan–protocatechualdehyde; DSF, disulfiram; ECM, extracellular matrix; EGCG, epigallocatechin gallate; GHK, glycyl-L-histidyl-L-lysine; GSH, glutathione; MOF, metal–organic framework; NPs, nanoparticles; PD, pharmacodynamics; PK, pharmacokinetics; PLA/Ch, polylactic acid/chitosan; ROS, reactive oxygen species; SCC, squamous cell carcinoma. Copper transporters: CTR1 and ATP7A/B; cuproptosis effectors: FDX1 and LIAS.

## Data Availability

No new data were created or analyzed in this study. Data sharing is not applicable to this article.

## Abbreviations

The following abbreviations are used in this manuscript:

3CL^PRO^	3C-like protease
DGG	Deacetylated gellan gum
DLAT	Dihydrolipoamide S-Acetyltransferase
DPPC	Dipalmitoylphosphatidylcholine
DSPE-PEG	Distearoylphosphatidylethanolamine-Poly(ethylene glycol)
FDA	Food and Drug Administration
FDX1	Ferredoxin-1
IC_50_	Inhibitory Concentration
MIC	Minimum Inhibitory Concentration
PAS	Amino-propanediol sebacate
PEG2000	Polyethylene glycol 2000
PLGA	Poly(lactic-co-glycolic acid)
PVA	Polyvinyl Alcohol
ROS	Reactive oxygen species
SH-PEG-OH	Thiol-PEG-Hydroxyl
SI	Selectivity Index
TEOS	Tetraethyl orthosilane
